# Disproportionate Alterations in the Anterior and Posterior Insular Cortices in Obsessive–Compulsive Disorder

**DOI:** 10.1371/journal.pone.0022361

**Published:** 2011-07-21

**Authors:** Aram Song, Wi Hoon Jung, Joon Hwan Jang, Euitae Kim, Geumsook Shim, Hye Yoon Park, Chi-Hoon Choi, Jun Soo Kwon

**Affiliations:** 1 Interdisciplinary Program in Neuroscience, College of Natural Sciences, Seoul National University, Seoul, Republic of Korea; 2 Department of Psychiatry, College of Medicine, Seoul National University, Seoul, Republic of Korea; 3 Department of Radiology, National Medical Center, Seoul, Republic of Korea; 4 Brain and Cognitive Sciences—World Class University Program, College of Natural Sciences, Seoul National University, Seoul, Republic of Korea; The University of Hong Kong, Hong Kong

## Abstract

**Background:**

Recent studies have reported that the insular cortex is involved in the pathophysiology of obsessive–compulsive disorder (OCD). However, specific morphometric abnormalities of the insular subregions remain unclear. In this study, we examined insular cortical volume to determine whether the volume of the anterior and posterior insular cortices of unmedicated OCD patients differed according to different symptom dimensions.

**Methods/Principal Findings:**

Using magnetic resonance imaging, we measured the gray matter volumes of the insular cortex and its subregions (anterior and posterior divisions) in 41 patients with OCD (31 drug-naïve and 10 non-medicated) and 53 healthy controls. Volumetric measures of the insular cortex were compared according to different OC symptoms. Enlarged anterior and reduced posterior insular cortices were observed in OCD patients. The insular volumetric alterations were more significant in OCD patients with predominant checking rather than cleaning symptoms when compared with healthy controls.

**Conclusions/Significance:**

Our results suggest the presence of unbalanced anterior and posterior insular volumetric abnormalities in unmedicated OCD patients and emphasize the distinct role of the insular cortex in different OC symptoms. We propose that the insular morphometric alterations may influence the modulation of interoceptive processing, the insular functional role, in OCD patients with different symptoms.

## Introduction

The insular cortex integrates multimodal sensory information and plays an important role in affective and cognitive processing. The insula is involved in autonomic regulations, including vestibular function and visceral sensory/motor inputs related to gustatory, olfactory, visual, auditory, and tactile data [1]. A recent review emphasized the role of the insular cortex in interoception, which is conceptualized as the sense that deals with the physiological condition of the entire body [2]. The insular cortex is anatomically separated into the anterior insular cortex (AIC) and the posterior insular cortex (PIC). Each of these insular subregions has a functionally distinct role in interoceptive processing. The PIC receives primary interoceptive signals from the thalamus via the lamina I spinothalamocortical pathway, and the AIC re-represents the signals with subjective feelings [2]. The PIC has been activated by various modalities combined with sensory information [3], [4], [5]. The AIC, on the other hand, has been related to subjective evaluations associated with the visceral sensations [3], [6], [7].

Recent imaging studies have demonstrated that the insular cortex is involved in the neural mechanisms underlying obsessive–compulsive disorder (OCD) [8], [9], [10]. The insula is located in the center of the brain, and its subregions have reciprocal connections with other brain regions in the fronto–striato–thalamo–cortical (FSTC) circuitry, which is the current pathophysiological model for OCD [11], [12], [13], including the orbitofrontal cortex (OFC), anterior cingulate cortex (ACC), dorsolateral prefrontal cortex (DLPFC), thalamus, and amygdala [1]. The AIC particularly has strong interaction with the OFC that its overactivation is commonly observed in OCD patients [14]. Husted et al. (2006) emphasized the OFC hyperactivity might arise from the AIC. In contrast, the PIC has been known to connect with temporal and parietal cortices [15]. Thus, it is conceivable that the afferent and efferent projections between components of the FSTC pathway and each insular subregion may be important in the modulation of interoceptive processing among OCD patients.

Altered interoception, associated with the insular cortex, could underlie the initiation of the anxiety that results in future aversive body states in anxiety-prone individuals [16]. OCD is an anxiety disorder described as intrusive thoughts and repetitive behaviors. The interoceptive exaggeration develops into a driving force [17] characterized by elevated anxiety, worried thoughts, and compulsive behaviors designed to attenuate the anxiety [16] in OCD patients. The AIC might provides the excessive prediction signals in symptom-relevant situations, based on the primary interoceptive information from the PIC. Numerous functional magnetic resonance imaging (fMRI) studies have documented that insular activities are differentially correlated with distinct OC symptoms [18]. Exaggerated feelings of disgust, which is implicated in the AIC role, were associated with cleaning symptoms in OCD patients [19], [20]. In addition, OCD patients with checking symptoms exhibited greater inability to tolerate erroneous conditions related to the insular activations than patients with cleaning symptoms [21], [22]. However, thus far, little about structural abnormalities in the insular subregions of OCD patients has been understood. In particular, no reported study has focused on the anatomical features of the insular subregions as a function of different OC symptoms.

The purpose of this study was to use a multidimensional perspective on OC symptoms to determine anterior and posterior insular morphometric alterations associated with OCD. Given the differences between the AIC and the PIC in terms of anatomical features and functional roles related to interoceptive processing, we hypothesized that the volume of each insular subregion would demonstrate different relationships with the predominant OC symptoms in OCD patients.

## Materials and Methods

### Ethics statement

This study was conducted according to the principles expressed in the Declaration of Helsinki. The Institutional Review Board at Seoul National University Hospital approved the current study. Participants provided written informed consent after the procedure had been fully explained.

### Participants

Forty-one patients with OCD (29 males: 23 drug-naïve and 6 non-medicated, 12 females: 8 drug-naïve and 4 non-medicated) were recruited from the OCD clinic at Seoul National University Hospital. Patients were diagnosed using the Structured Clinical Interview for DSM-IV (SCID) [23], based on DSM-IV criteria for OCD. Annett's questionnaire [24] and the Korean version of the Wechsler Adult Intelligence Scale (K-WAIS) were used to measure handedness and estimated IQ, respectively. Fifty-three age-, gender-, and IQ-matched healthy controls (41 males, 12 females) also participated after recruitment through newspaper or Internet advertisements. We screened control participants using the SCID Non-Patient Version [25] to exclude a lifetime history of DSM-IV axis I disorders. Exclusion criteria for all participants included major depressive disorder, mental retardation, significant head injury, seizure disorder, substance abuse or dependence, and history of psychotic disorders, such as bipolar disorder and schizophrenia.

The patient group was composed of 31 drug-naïve and 10 non-medicated for a period longer than 4 weeks before the study. Six patients with OCD had comorbid conditions including personality disorders (*n* = 4: three with obsessive–compulsive personality disorder, one with schizotypal personality disorder), panic disorder (*n* = 1), and tic disorder (*n* = 1). No additional comorbid axis I DSM-IV diagnosis was observed in the remaining 35 patients with OCD. We classified OCD patients according to the predominant four symptom dimensions: obsessions/checking (*n* = 25), contamination/cleaning (*n* = 14), symmetry (*n* = 2), and hoarding (no patient) [26]. We measured OC symptom severity using the Yale–Brown Obsessive–Compulsive Scale (Y–BOCS) [27], [28] and assigned a score of 0 (absent), 1 (mild), or 2 (prominent) for each of the clinical dimensions. The highest score was used as the overall score for that dimension. We assessed levels of depression and anxiety using the Beck Depression Inventory (BDI) [29] and the Beck Anxiety Inventory (BAI) [30], respectively.

### MRI acquisition and processing

Magnetic resonance images were acquired using a 1.5-T Magnetom Avanto scanner (Siemens, Erlangen, Germany) at the National Medical Center. The scanning parameters of the three-dimensional T1-magnetization prepared rapid gradient echo (MPRAGE) were as follows: 0.9 mm axial slices, echo time = 4.76 ms, repetition time = 1160 ms, flip angle = 15°, matrix size = 256×256, and FOV = 230 mm, no gap. To increase the signal-to-noise ratio, we scanned all participants three times and obtained the average image from the three scans. The scanner was calibrated monthly with the same phantom to ensure stability of the measurements. All images were collected using an image-processing software package, ANALYZE (ver. 9.0; Mayo Foundation, Rochester, MN). Images were resampled to 0.9 mm^3^ iso-voxels and spatially realigned to correct head movement, based on the axis of the anterior–posterior commissural line. We applied intensity corrections with an iterative expectation-maximization algorithm that estimated the non-homogeneity of image intensity. The semi-automated region growing method removed tissues exterior to the brain images. The intracranial volume (ICV) was calculated by summing the segmented subtotals of gray matter, white matter, and cerebrospinal fluid volumes to correct for differences in head size.

### Insular cortex measurements

**Figure 1:**
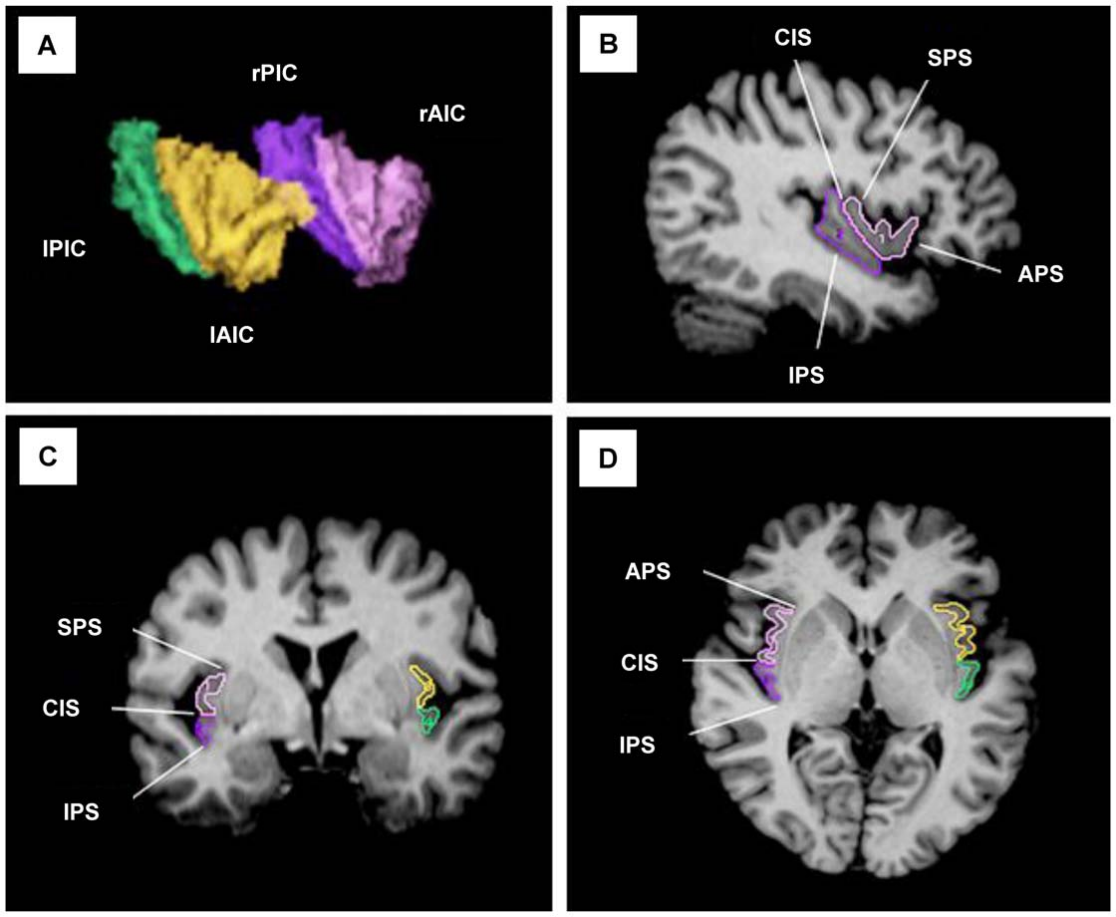
Two-and three-dimensional images of the anterior and posterior insular cortices. (**A**) A 3D-reconstructed image of the insular cortex. (**B**) Sagittal, (**C**) coronal, and (**D**) axial views of the anterior insular cortex (pink on right side and yellow on left side) and the posterior insular cortex (purple on right side and green on left side). APS, anterior peri-insular sulcus; CIS, central insular sulcus; IPS, inferior peri-insular sulcus; SPS, superior peri-insular sulcus; rAIC, right anterior insular cortex; rPIC, right posterior insular cortex; lAIC, left anterior insular cortex; lPIC, left posterior insular cortex.

The insular cortex was manually traced on consecutive coronal slices using the region of interest (ROI) module of ANALYZE. The drawing of ROIs (left AIC and PIC, right AIC and PIC) was performed without knowledge of the diagnosis, gender, and hemisphere. We followed the same criteria described in a previous anatomical study [31]. The anterior peri-insular sulcus (APS), inferior peri-insular sulcus (IPS), and superior peri-insular sulcus (SPS) differentiate the insular cortex from other cortical areas. The SPS and IPS define the dorsal and ventral boundaries of the insular cortex, and the APS is the inferior boundary between the AIC and OFC. The fusion of the SPS and APS was considered to be the most caudal point of the insula, and that of the SPS and IPS was defined as the rostral point of the insula. The insular cortex is separated into subregions by the central insular sulcus (CIS), which is regarded as the landmarks for the inferior and superior boundaries of the AIC and PIC, respectively; the AIC is composed of the three short gyri and the PIC is composed of the two long gyri. The CIS was well defined (96.8% of participants) in this study. When the CIS was not clear, we used alterative landmark defined reliably as 1 slice caudal to appearance of mamillary bodies for anterior boundary of the PIC [32]. The insular ROI volume was automatically calculated by summing successive images using the ANALYZE program. On average, 60 coronal slices were obtained for the ROIs, and the three-dimensional reconstructed images of the insular cortices are described in Figure 1A.

All insular ROIs were investigated by one rater (A.S.), and 20 cases were randomly selected to determine the inter-rater reliability of the measurements. Two raters (A.S. and W.H.J.) independently conducted the tracing process while blinded to diagnosis, gender, and hemispheric information. Pearson's correlation coefficients were 0.93 and 0.96 for the right and left AIC, respectively, and 0.92 and 0.94 for the right and left PIC, respectively.

### Statistical analyses

Demographic and clinical characteristics of OCD patients and healthy controls were compared using independent sample *t*-tests and chi-square tests. Intracranial volumetric differences between groups were examined with an analysis of covariance (ANCOVA) using age as the covariate. Data were analyzed in three steps. First, absolute insular volumetric measurements were analyzed using multivariate ANCOVA (MANCOVA), with diagnosis as a between-group factor and age, ICV, and BDI score as the covariates. Second, relative insular volumes ([absolute volume/ICV]×100) of the groups were compared using age and BDI score as the covariates for the MANCOVA. Third, we evaluated the ratios of the AIC and PIC volumes to the volume of the entire insular cortex ([subregional volume/regional volume]×100) using MANCOVA, with diagnosis as a between-group factor and ICV and BDI score as the covariates. The asymmetry index was calculated with the formula ([right-left]/[right+left]×100) [33] and analyzed using one-way analysis of variance (ANOVA) for the group comparison of insular lateralization. To explore the relationship of insular volume ratios to the dimensional aspects of OCD, we performed MANCOVA with ICV and BDI score as covariates followed by *post-hoc* Tukey's honestly significant difference (HSD) test. We excluded OCD patients with the symmetry dimension (*n* = 2) from the comparison because checking (*n* = 25) and cleaning (*n* = 14) symptoms were predominant among the 41 OCD patients.

Pearson's correlation coefficients were used for evaluating the correlations between all investigated insular volumes and clinical measures (illness duration, BDI/BAI, and Y–BOCS scores). All statistical analyses were performed as two-tailed or *post-hoc* using the SPSS software (ver. 12.0 for Windows; SPSS, Chicago, IL). The significance level was set at *P*<0.05 for all the statistical results.

## Results

We found no significant difference between groups in terms of age, gender, handedness, education, and estimated IQ, whereas OCD patients showed higher BDI and BAI scores than did healthy controls (*P*<0.001). Fifteen patients (10 males and 5 females) scored over 16 in BDI self-reports, but no patient had been diagnosed with major depressive disorder using SCID I. Table 1 summarizes the demographic and clinical characteristics of each group.

**Table 1:** Demographic and clinical characteristics of patients with obsessive–compulsive disorder and healthy controls^a^.

	OCD patients	Healthy controls	Statistics	
Variable	(n = 41)	(n = 53)	χ^2^/*t*	*P*
Gender (male/female)	29/12	41/12	0.534	0.484
Handedness (right/left)	38/3	53/0	4.006	0.080
Age (years)	23.80±6.24	23.91±2.84	0.096	0.924
Education (years)	13.93±3.14	14.40±1.29	0.900	0.373
Estimated IQ	109.27±13.60	113.17±10.71	1.557	0.123
Illness duration (years)	6.70±5.74	-	-	-
BAI score^b^	17.51±12.74	5.28±5.53	−5.741	<0.001***
BDI score^b^	15.51±10.14	4.66±6.00	−6.078	<0.001***
Y–BOCS score				
Obsessive score	11.20±3.49	-	-	-
Compulsive score	9.98±4.66	-	-	-
Total score	21.17±5.97	-	-	-

BAI, Beck Anxiety Inventory; BDI, Beck Depression Inventory; IQ, intelligence quotient; OCD, obsessive–compulsive disorder; Y–BOCS, Yale–Brown Obsessive Compulsive Scale.

a Values are presented as mean±SD otherwise stated.

b OCD patients showed significantly higher scores than healthy controls in independent sample *t*-test.

***P<0.001.

### Group comparisons of insular cortical volumes

**Figure 2:**
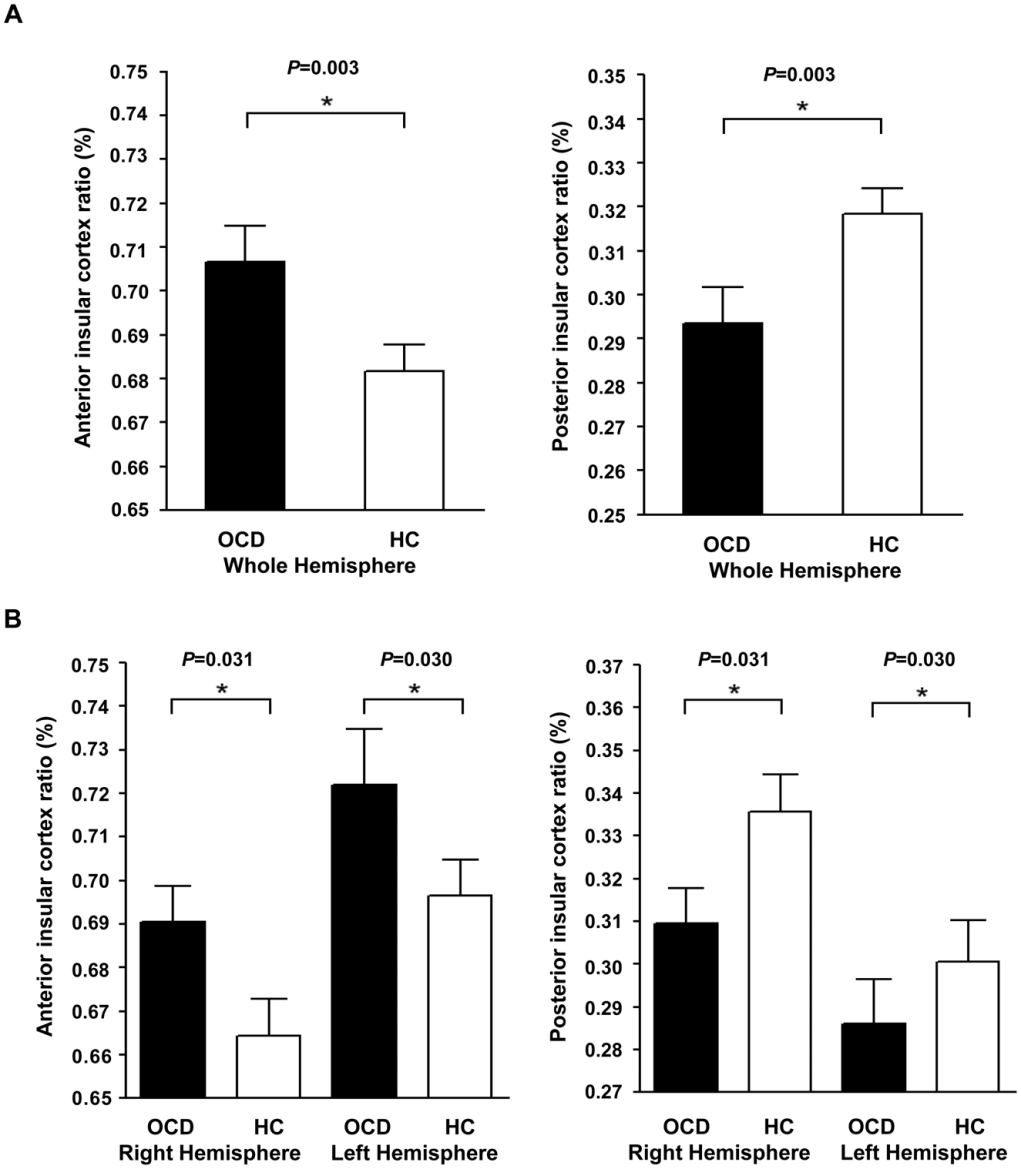
Asymmetric relation between anterior and posterior insular cortical volumes. The bar graph indicates the ratios of the anterior and posterior insular volumes to the entire insular volume. Each ratio was calculated for the whole brain (A) and for each hemisphere (B) in obsessive-compulsive disorder patients (n = 41) and healthy controls (n = 53). Error bars indicate SEM. HC, healthy control; OCD, obsessive–compulsive disorder.

Absolute and relative volumetric comparisons are presented in Table 2. The ANCOVA revealed no significant difference in ICVs between groups (*P*>0.05). Both the absolute (*F* = 7.055, *P* = 0.009) and relative (*F* = 6.811, *P* = 0.011) volumes of the AIC were enlarged in OCD patients compared with those in healthy controls. In contrast, the absolute (*F* = 4.245, *P* = 0.042) and relative (*F* = 4.966, *P* = 0.028) volumes of the PIC were reduced in OCD patients compared with those in healthy controls. The MANCOVA also revealed significant group differences in asymmetric relation between the AIC and PIC volumes (Fig. 2). The AIC portion of the entire insular cortical volume was significantly increased and that of the PIC was significantly decreased in OCD patients compared with healthy controls (*F* = 9.449, *P* = 0.003) in both hemispheres (right: *F* = 4.800, *P* = 0.031; left: *F* = 4.874, *P* = 0.030). The asymmetry indexes of the insular lateralization did not differ significantly between groups (*P*>0.05).

**Table 2:** Absolute and relative volumes^a^ of the insular cortex in obsessive–compulsive disorder and healthy controls^b^.

	OCD patients	Healthy controls	Analysis of covariance	
	(n = 41)	(n = 53)		
Brain region and volume type	mean±SD	mean±SD	*F*	*P*
Intracranial volume, mL^c^	1529±110	1519±104	0.177	0.675
Insular cortex				
Absolute (mL)	9.34±1.23	9.25±0.95	1.403	0.239
Relative (%)	0.61±0.06	0.61±0.05	1.004	0.319
Anterior^d^				
Absolute (mL)	6.61±1.08	6.31±0.79	7.055	0.009**
Relative (%)	0.43±0.06	0.42±0.05	6.811	0.011*
Posterior^d^				
Absolute (mL)	2.74±0.57	2.95±0.47	4.245	0.042*
Relative (%)	0.18±0.03	0.19±0.03	4.966	0.028*

OCD, obsessive–compulsive disorder.

a Calculated as follows: (absolute insular volume/intracranial volume)×100.

b Age and Beck Depression Inventory score were used as the covariates for both absolute and relative insular volumes. Intracranial volume was added as a covariate for absolute insular volume.

c Age was used as a covariate.

d OCD patients showed significantly larger anterior and smaller posterior insular cortical volumes than healthy controls (Analysis of covariance, *P*<0.05).

*P<0.05,

**P<0.01.

### Differences in symptom dimensions according to insular cortical volume

Table 3 summarizes the insular cortical measurements of OCD patients with predominantly checking and cleaning symptoms and those of healthy controls. The three groups differed in the ratios of the AIC and PIC volumes to the entire insular volume (*F* = 5.450, *P* = 0.006). *Post-hoc* analyses (Tukey's HSD test) revealed increased AIC and decreased PIC portions in OCD patients with predominant checking (*P* = 0.014) but not cleaning (*P*>0.05) symptoms compared with those in healthy controls. This finding was more significant in the right (*P* = 0.031) than the left (*P*>0.05) hemisphere. No significant differences in the volumes of the whole insular cortex were found among the groups (*P*>0.05).

**Table 3:** Differences in symptom dimensions of insular volume ratio in obsessive–compulsive disorder and healthy controls^a^.

	OCD checking	OCD cleaning	Healthy controls	Analysis of covariance^b^			
	(n = 25)	(n = 14)	(n = 53)	Group effect		*Post-hoc* tests	
Brain region	mean±SD	mean±SD	mean±SD	*F*	*P*	Group comparision	*P*
IC, mL	9.56±1.37	9.05±0.94	9.25±0.95	1.606	0.208	NA	NA
AIC (%)^c^	71.37±5.41	69.53±5.05	68.09±4.21	5.450	0.006*	check>clean = HC	0.014*
PIC (%)^c^	28.63±5.41	30.47±5.05	31.91±4.21	5.450	0.006*	check<clean = HC	0.014*
Right IC, mL	4.83±0.78	4.61±0.50	4.66±0.52	1.290	0.259	NA	NA
AIC (%)^c^	70.16±5.76	67.13±3.77	66.51±6.32	3.507	0.034*	check>clean = HC	0.031*
PIC (%)^c^	29.84±5.76	32.87±3.77	33.49±6.32	3.507	0.034*	check<clean = HC	0.031*
Left IC, mL	4.72±0.61	4.43±0.47	4.59±0.46	1.728	0.192	NA	NA
AIC (%)	72.59±8.13	71.91±9.05	69.71±5.88	2.573	0.082	NA	NA
PIC (%)	27.41±8.13	28.09±9.05	30.29±5.88	2.573	0.082	NA	NA

AIC, anterior insular cortex; HC, healthy control; IC, insular cortex; NA, not applicable; OCD, obsessive-compulsive disorder; PIC, posterior insular cortex.

a Calculated as follows: (insular subregional volume/whole insular volume)×100.

b Intracranial volume and Beck Depression Inventory score were used as the covariates.

c Tukey's honestly significant difference test was used for *post-hoc* test.

*P<0.05.

### Correlation analyses

Negative correlations were observed between the absolute AIC volume and BDI score (*r* = −0. 365, *P* = 0.019) and the relative AIC volume and BDI score (*r* = −0.316, *P* = 0.044) among OCD patients. No significant correlation between the ratios of the AIC and PIC volumes to the entire insular volume and BDI scores (*P*>0.05) was found in any group. To eliminate the effects of depression on insular volumes, we controlled for the BDI scores as the covariate in all volumetric measurements of the insular cortex. In contrast, BAI score showed no significant correlation with the volume of the insular cortex in any group. Clinical variables, including illness duration, subscores (obsessions and compulsions), and total scores on the Y–BOCS, were not significantly correlated with the volumes of the insular cortex in OCD patients.

## Discussion

The present study was designed to determine whether dissimilar morphometric alterations in the anterior and posterior insular subregions would be observed in unmedicated OCD patients. OCD patients showed enlarged AIC and reduced PIC volumes compared with those of healthy controls. Interestingly, the pattern of these morphometric abnormalities was significant in those with predominant checking symptoms, who showed disproportionate alterations in AIC and PIC volumes, but not in those with predominant cleaning symptoms.

### Unbalanced morphometric alterations of the insular subregions in OCD

Our results correspond well with previous studies reporting enlarged AIC volume [10], [34] and reduced PIC volume [9] in OCD patients. Additionally, reports that the volume of the entire insular cortex of OCD patients was intact [33] are consistent with our findings. However, reduced volume in the insulo–opercular region [9] and increased volume in the right insular cortex have been reported in OCD patients [35]. This difference between prior studies and the present research may be due to varying study designs and methodological differences. First, most structural studies included both medicated and drug-naïve patients. A previous study reported that antidepressant drugs such as selective serotonin reuptake inhibitors (SSRIs) influence on the thalamic volumes in OCD patients [36]. The insular activation is also known to be influenced by SSRIs, which are commonly used in the pharmacological treatment of OCD patients. The SSRIs effect on the insular cortex for attenuating the activation during emotional processing, including regulation of anxiety which is important for OCD [37]. To eliminate the medication effect on the insular volume of OCD patients, three-quarters of the current sample was drug-naïve and one-quarter was non-medicated (>4 weeks). Second, to date, insular volumetric studies of OCD have been performed using automatic voxel-based morphometry (VBM) analyses, which may influence the results in the adjacent cortical areas of the insular cortex. VBM results are sensitive to the kernel size and may not detect individual variability in the small and localized gray matter volumes in course of spatial normalization. Though ROI creation is one of the highly labor-intensive approaches, it could ameliorate problems of comparing data across subject due to eliminate inaccuracies which arise from normalization of individual brain anatomy to a reference brain. Thus, the conventional manual ROI approach remains the gold standard for structural exploration, especially for the insular region, which is located deep inside the brain.

### Pyramidal shaped spindle neurons in the insular cortex

Morphometric abnormalities of the insular cortex in OCD may depend on disrupted brain organization during neurodevelopment. Our data showed disproportionate alterations in the AIC and PIC volumes of OCD patients. The insula is the first cortical region to develop in the human fetal brain, and the anterior and posterior portions differentiate from each other at between 27 and 28 gestational weeks [38]. The dorsal part of the PIC contains sensory representations of small-diameter signals, which constitute primary interoceptive images of homeostatic states, from the ventro–posterior–medial thalamic nucleus [2]. Given that interoceptive information is relayed from the PIC to the AIC, declining neuronal growth in the PIC may lead to compensatory activation in the AIC, such as exaggerated interoception, with increased AIC volume in OCD patients.

The AIC contains the unique large spindle neurons known as Von Economo Neurons (VENs) [39], [40]. The VENs are specially located in layer V of the AIC, ACC, and DLPFC, and are present only in hominoid primates [40]. Excessive survival of the spindle neurons in the AIC, a consequence of unsuccessful programmed cell death, may be an etiologically early neural substrate of neuropsychiatric conditions, notably OCD [1]. The VENs integrate representations of emotional moments and behavior [2] and possibly underlie the exaggeration of interoception or conscious awareness of visceral activity in OCD [39]. This altered interoception may be attributable to the rapid transmission of signals to adjacent nerve cells from VENs [39], [40]. The mismatch between current and anticipated body states may lead to a prediction-error signal that acts as a strong impulse for immediate changes in behavioral responses until the error is eliminated [41]. This mechanism could result in the habitual way of interpreting incoming sensory information [17] associated with the obsessive evaluations of internal states observed in OCD patients. The repetitive exaggerated prediction-error signals from the AIC to other brain regions may give rise to the compulsory behaviors characterized by high physiological arousal that are observed in OCD patients.

### Distinct role of the insular cortex in dimensional aspects of OCD

A general consensus holds that OCD is a clinically heterogeneous disorder, mediated by a distinct neural network and structural substrates of the symptom dimensions of OCD [12], [18], [42]. Recent default mode network study (DMN) found that the opposite direction of the DMN was observed in predominant checking and cleaning symptom dimensions during the resting state [42]. It has been suggested that the right AIC plays an important role in brain network level [43]. One remarkable finding of this study was that the insula was differentially associated with different dimensional aspects of OCD. Among OCD patients in the predominant checking group, but not the predominant cleaning group, showed a significantly increased AIC portion of the whole insular cortex and a significantly decreased PIC portion of the whole insular cortex when compared with healthy controls. This result was more significant in the right than in the left insular cortex. The present study could support an asymmetric role of the right insular cortex as well as the existence of different symptom dimensions in OCD.

The neural mechanism underlying interoception is more dominant in the right hemisphere [16], [40], meaning that the right insular cortex is more influential in providing a detailed representation of the internal state [31]. A developed right AIC has a distinct role in emotional aspects of interoception - bodily arousal responses are basis in emotional experiences and guide preconscioius adaptive behavior - [44] and renders somatic states more accessible to integration with experience-based biasing signals from the body [39]. The higher prediction-error signals emanating from the developed AIC would guide biased decision making with emotional arousal and individual preferences in uncertain environments [45]. Enhanced AIC activation was correlated with increased emotional salience state in anxiety-prone individuals using risk-processing tasks [46], [47]. OCD patients with predominant checking symptoms have described a high degree of intolerance of uncertainty [21]. The inability to tolerate uncertain situations may trigger the anxiety and compulsive behaviors in OCD patients with checking symptoms. It has been emphasized that scores on intolerance of uncertainty could quantify the significance of the prediction-error signals related to the insular cortex [21]. Consequently, compulsive checking rituals may be triggered in OCD patients to attenuate the anxiety deriving from the exaggerated signals originating in the enlarged AIC.

Nevertheless, the current findings should be considered in the context of the results of prior studies on insular dysfunction among OCD patients with predominant cleaning symptoms, patients who tend to experience strong feelings of disgust under symptom-relevant conditions [48]. The insula has been regarded as occupying a functional role in the processing of disgust in OCD [15]. Disgust is a predictor of compulsive checking and cleaning behaviors in OCD patients [49], and a hoarding compulsion may be associated with reduced sensitivity to disgust [18]. Evidence for the relationship between disgust and the dimensional view of OCD has underscored the role of extended feelings of disgust in not only disease-avoidance but also self-disgust and guilt [50]. Thus, abnormalities in the processing of disgust in the insular cortex seem to be a general characteristic of OCD rather than a specific feature of the cleaning-symptom dimension.

### Limitations

Several limitations of this study need to be mentioned. First, depression and anxiety levels were significantly higher in OCD patients, and these may have confounding effects on the current results. Thus, we attempted to examine the influences of these factors on insular volumes by evaluating the correlation analyses. Negative correlations were observed between BDI scores and insular cortical volumes, whereas no significant correlation was observed between BAI scores and insular volumes in OCD patients. Although no patient suffered with comorbid major depressive disorder, we used BDI scores as the covariate for all the insular volumetric measurements to increase the validity of the present study. Second, a greater number of male than female participants were included in this study. It has been suggested that gender may contribute to the clinical and biological heterogeneity of OCD [51]. However, no significant impact on the results was observed when gender was controlled for as the covariate.

### Conclusions

To our knowledge, this is the first reported morphometric study to investigate disproportionate alterations in the AIC and PIC volumes of OCD patients in terms of the heterogeneous aspects of OC symptoms. It has been suggested that the enlarged AIC and the reduced PIC may lead to exaggerated prediction-error signals that bias the affective and cognitive processes in OCD patients. Dissimilar morphometric alterations of the insular subregions may play important roles in the modulation of interoceptive processing in OCD, particular among patients with predominant checking symptoms. Further research is necessary to identify whether the structural abnormalities in the insular subregions play a causal role in OC symptoms via altered interoception and to examine the implications of the findings in this regard for the development of novel therapies for OCD patients.
